# Innate immune response in neuronopathic forms of Gaucher disease confers resistance against viral-induced encephalitis

**DOI:** 10.1186/s40478-020-01020-6

**Published:** 2020-08-24

**Authors:** Sharon Melamed, Roy Avraham, Deborah E. Rothbard, Noam Erez, Tomer Israely, Ziv Klausner, Anthony H. Futerman, Nir Paran, Einat B. Vitner

**Affiliations:** 1 Department of Infectious Diseases, Israel Institute for Biological Research, P.O. Box 19, 7410001 Ness-Ziona, Israel; 2 Department of Biomolecular Sciences, Weizmann Institute of Science, Rehovot, Israel; 3 Department of Applied Mathematics, Israel Institute for Biological Research, P.O. Box 19, 7410001 Ness-Ziona, Israel

**Keywords:** Gaucher’s disease, Alphaviruses, Sindbis virus, Encephalitis, Glucosylceramide, Brain inflammation, Astrocytosis, Microgliosis

## Abstract

Both monogenic diseases and viral infections can manifest in a broad spectrum of clinical phenotypes that range from asymptomatic to lethal, suggesting that other factors modulate disease severity. Here, we examine the interplay between the genetic neuronopathic Gaucher’s disease (nGD), and neuroinvasive Sindbis virus (SVNI) infection. Infection of nGD mice with SVNI had no influence on nGD severity. However, nGD mice were more resistant to SVNI infection. Significantly different inflammatory responses were seen in nGD brains when compared with SVNI brains: the inflammatory response in the nGD brains consisted of reactive astrocytes and microglia with no infiltrating macrophages, but the inflammatory response in the brains of SVNI-infected mice was characterized by infiltration of macrophages and altered activation of microglia and astrocytes. We suggest that the innate immune response activated in nGD confers resistance against viral infection of the CNS.

**Electronic supplementary material:**

The online version of this article (10.1186/s40478-020-01020-6) contains supplementary material, which is available to authorized users.

## Introduction

Brain inflammation is a common characteristic of many disorders of the central nervous system (CNS). Irrespective of whether a disease is genetic or infectious in origin, activation of the brain’s immune response is normally present [66]. The CNS resident immune system is comprised mainly of innate immune cells, with microglia and astrocytes the key components. However, under certain pathological conditions, peripheral innate and adaptive immune cells, including monocytes, neutrophils, T cells and B cells, can enter the CNS [69]. The inflammatory response can be chronic or acute, and the outcome of the activation may be beneficial, deleterious, or neutral.

Infection of the CNS is a major global healthcare concern, and is associated with high morbidity and mortality [38, 71]. Neuroinfectious diseases may be associated with acute changes in mental and motor function, followed by chronic neurological dysfunction which can persist long after recovery from the infectious event.

In neuroinfectious diseases, the innate immune response is critical for limiting pathogen invasion of the CNS and essential to mediate host resistance. However, if the response fails to eliminate the aggressor, inflammation may lead to progressive disease and ultimately death. Persistent activation of the immune response in the CNS has been implicated as a cause of long-term neurological impairment [80]. Thus, delineating between beneficial and detrimental signaling pathways in the inflammatory response against the pathogen is crucial for developing a therapeutic strategy. Similar to neuroinfectious diseases, the innate immune response is also associated with genetic neurodegenerative diseases despite their different etiologies. However, while the central role of the inflammatory response in infectious disease is well established, its role in genetic diseases is not fully understood. In contrast to infectious diseases where immune activation is resolved by elimination of infectious agents, the immune response stimuli persist in genetic diseases and cannot be resolved, resulting in chronic inflammation. Over the past few years, many studies have attempted to elucidate the role of innate immune activation in genetic neurodegenerative diseases such as Alzheimer’s disease [15], Parkinson’s disease (PD) [70], and lysosomal storage diseases (LSDs) [37, 56, 79, 81, 90]. Studies in mouse models, epidemiologic studies, and meta-analysis suggest a neutral to moderately detrimental role of the innate immune response in neurodegenerative disease pathology, as observed by the limited or non-effectivity of nonsteroidal anti-inflammatory drugs (NSAIDs) and by genetic knock-out of immune pathways [37, 56, 79].

Gaucher disease (GD), an autosomal recessive metabolic disorder caused by mutations in the glucocerebrosidase gene (*GBA*), is the most common of the LSDs [24]. *GBA* encodes for lysosomal glucocerebrosidase (GCase) (EC 3.2.1.45; acid beta-glucosidase) which catalyzes the degradation of glucosylceramide (GlcCer). Mutations in *GBA* present a wide spectrum of clinical manifestations [77]; homozygous and heterozygous mutations in *GBA* numerically constitute the most prominent risk factor for Parkinson’s disease (PD), and homozygous mutations in *GBA* result in GlcCer accumulation, leading to GD pathology.

GD is divided into three broad subgroups based upon the presence or absence of neurological involvement: type 1 (non-neuronopathic), type 2 (acute neuronopathic), and type 3 (subacute neuronopathic). The clinical manifestations of type 2 GD (OMIM #230900) can range from hydrops fetalis to the collodion baby phenotype. Uniformly, there is rapid progression with severe neurodegeneration, leading to death in infancy or early childhood [48, 78]. The hallmark clinical abnormality seen in type 3 GD (OMIM #2301000) consists of markedly slow horizontal saccades [25, 72]. Both type 2 and 3 GD, the neuronopathic forms of Gaucher disease (nGD), are characterized by region-specific gliosis. This gliosis is observed in CA2–CA4 of the hippocampus, layer III and V of the parietal cortex, and layer IVb of the occipital cortex. Neuronal loss is also noted in these regions in patients with neuronopathic GD [92]. The genetic murine model which recapitulates many features of human nGD is the *Gba*^flox/flox^; nestin-Cre mouse [19]. This murine model exhibits microglial activation and astrogliosis prior to disease manifestation, which is tightly correlated with neuron loss [22, 88]. Interestingly, these pathological events occur in specific brain areas (such as cortical layer V, also shown to be affected in human nGD brain) [92], while other brain regions remain unaffected even at late stages of the disease [21, 22]. The *Gba*^flox/flox^; nestin-Cre model has proven useful in nGD research, but its limited lifespan and symptom severity restrict its usefulness. Injection with the irreversible GCase inhibitor conduritol B-epoxide (CBE) [39] has also proven of great use, particularly since CBE crosses the blood–brain barrier (BBB) [82]. A comparison between *Gba*^*flox*/flox^; nestin-Cre mice and CBE-treated mice, indicates a remarkable overlap in gene expression profile changes, and a similar pattern of brain pathology [86]. Indeed, both murine models recapitulate region-specific pathology, activation of astrocytes and microglia, and neuronal loss [86, 91]. Moreover, similar innate immune activation pathways, including substantial activation of the type I interferon (IFN) response is evident in both models [22, 86, 89].

Milder *GBA* mutations are associated with non-nGD, while homozygosity for more severe mutations generally leads to nGD, although genotype–phenotype correlation is poor [20, 27]. Despite being a monogenic disorder, other factors such as genes, epigenetic modifiers, or environmental influences, may modulate disease severity [40]. The existence of identical twins with differing levels of disease severity [48] implies a role for non-genetic factors in modulation of disease outcome.

Similar to genetic diseases, viral infection can manifest by a broad spectrum of clinical phenotypes, ranging from asymptomatic to lethal. This has been observed in many viral infections such as Zika virus [75], West Nile virus (WNV) [83], Cytomegalovirus (CMV) [16], and Epstein-Barr virus (EBV) [63]. Indeed, infection with each of these viruses has been associated with brain inflammation.

The continuing spread of CNS-associated viral infection, combined with the lack of specific avenues to combat or prevent infection, imparts a pressing need to identify the viral and host processes which affect the outcome of those infections.

Here, we studied the interplay between a genetic neurodegenerative disease and viral infection of the CNS. Recently, activation of type 1 IFN response was identified in mouse models of nGD [89], which could, in principle, limit viral infection. Alternatively, further activation of the immune response by viruses may accelerate nGD progression and result in a more severe manifestations. We examined the response of nGD murine models to neuroinvasive Sindbis virus (SVNI), a prototypic member of the Alphavirus genus that has been used to study the pathogenesis of acute viral encephalitis in mice for many years [18, 44, 58]. Studies with neuroinvasive Sindbis virus demonstrated that it infects neurons and that recovery from infection requires noncytolytic clearance to avoid damaging these irreplaceable cells. In mature mice, the populations of neurons most susceptible to infection are in the hippocampus and anterior horn of the spinal cord. Hippocampal infection leads to long-term memory deficits in mice that survive, while motor neuron infection can lead to paralysis and death. Neuronal death is immune-mediated, rather than a direct consequence of viral infection, and associated with entry and differentiation of pathogenic T helper 17 cells in the nervous system [30]. Antiviral antibodies and interferon-γ have major roles in clearance with a likely role for both IgM and IgG antibodies [31].

We demonstrated that nGD murine models are more resistant to infection with SVNI. The resistance of nGD mice is most likely due to properties of the innate immune response that are triggered in nGD, in which the activated cell types are different from the immune response to SVNI. Accordingly, we identified innate immune pathways that may confer the resistance to viral infection of the CNS. These pathways may facilitate a new avenue of therapeutic intervention for viral infections.

## Materials and methods

### Mice

*Gba*^flox/flox^ mice were previously described [19]. *Gba*^flox/flox^ mice were crossed with *Gba*^flox/+^; nestin-Cre mice to generate *Gba*^flox/flox^; nestin-Cre mice and *Gba*^flox/+^; nestin-Cre mice, which served as healthy controls. Genotyping was performed by PCR using genomic DNA extracted from mouse tails or embryonic brains. Both male and female mice were used. The colony was maintained in the experimental animal center of the Weizmann Institute of Science. Animals were transferred to the Israeli Institute for Biological Research 2 days prior to viral-infection.

C57BL/6JOlaHsd mice were purchased from Envigo (Harlan). *Ifnar*^–/–^ mice purchased from The Jackson laboratory (number 32045). All mice were on the C57BL/6 background and maintained on a 12 h light/dark cycle, with food and water provided ad libitum. Mice were housed in microisolator cages with no more than 5 mice per cage and all experiments used littermate controls. All animals were maintained in specific-pathogen-free conditions and handled according to protocols approved by the Institutional Animal Care and Use Committee regulations, as per international guidelines. Animal work involving infectious virus was performed in the physical containment level 2 laboratory of the Israeli Institute for Biological Research.

### Viruses

The isolation and phenotype of SVNI has been described [55]. Briefly, a strain of Sindbis virus (SV), isolated from mosquitoes in Israel, was used as a source of SVNI. Serial passage of SV in suckling and weanling mouse brain was used to generate SVNI, a Sindbis virus which is both neurovirulent and neuroinvasive. Virus stock, prepared on Vero cells and stored in aliquots at − 70 °C, was used in all studies.

The original strain of WNV was isolated in Israel in 1952 from the blood of a human patient during the febrile phase of the disease [28]. After isolation, it was passaged several times in suckling mice brains and Vero cells. Virus stock prepared on BHK-21 cells and stored in aliquots at − 70 °C, was used in all studies.

### Conduritol B-epoxide (CBE) treatment

CBE (Calbiochem Millipore), an irreversible GCase inhibitor [39], was dissolved in phosphate-buffered saline [PBS] and injected intraperitoneally (i.p.) daily at a concentration of 50 mg/kg body weight from postnatal day 13.

### Viral infection in vivo

All mice were infected by i.p. injection of three lethal doses, 50% (3LD50). LD50 was initially pre-determined for every mouse strain and age.

16-days old *Gba*^flox/flox^;nestin- Cre mice and *Gba*^flox/WT^; nestin-Cre mice were infected with 5 pfu SVNI. *n* = 4 for control*, n* = 10 for SVNI*, n* = 3 for *Gba*^−/−^, *n* = 4 for *Gba*^−/−^ + SVNI.

21 day-old C57BL/6 mice were infected with 30 pfu SVNI (n = 8 mice/group) or 10 pfu WNV (n = 8 mice/group). *Ifnar*^−/−^ were infected with 5 pfu SVNI (n = 5–6 mice/group). Prior to viral infection, mice were injected daily i.p. with 50 mg/kg of CBE starting from day 13 of age. Mice were weighed and monitored daily.

### Enzyme-linked immunosorbent assay (ELISA)

Serum levels of anti-SVNI antibodies were determined by ELISA. ELISA plates were coated with 4.5 × 10^7^ pfu/ml of inactivated Sindbis virus in carbonate-bicarbonate buffer (C-3041; Sigma-Aldrich, St. Louis, MO) and incubated overnight at 4 °C. Plates were then washed three times with PBS-T (phosphate-buffered saline [PBS] containing 0.05% [vol/vol] Tween 20) and blocked for 1 h with PBS-2% bovine serum albumin (BSA)-0.05% Tween 20 at 37 °C. After three washes, mouse serum, diluted 1:100 in PBS containing 1% BSA, was applied for another hour, and then detected by alkaline phosphatase-anti-mouse immunoglobulin G (whole molecule) antibody (A-4312; Sigma). Values of at least twice the background signal (serum of uninfected mice) were considered positive. *n *= 4 for control, *n *= 5 for SVNI, *n *= 3 for CBE, *n *= 7 for CBE + SVNI.

### Activity of caspase-3/7

Caspase-3/7 (caspase 3 and 7 share the same substrate), were assayed using a Caspase-Glo assay kit (Promega). *n *= 3 for control and CBE + SVNI, n = 4 for SVNI and CBE.

### Viral genome quantification

Quantification of viral plus strand RNA was performed as previously described [64]. Total RNA was isolated from homogenized brain tissue using an RNeasy mini kit (Qiagen, Hilden, Germany) according to manufacturer’s instructions. cDNA was synthesized using Enhanced Avian Reverse Transcriptase [eAMV™ RT] (Sigma, A4464) with a SINV-specific primer (SINV9899R 5′ AGCATTGGCCGACCTAACGCAGCAC 3′ for the cDNA synthesis of plus strand RNA). Real time PCR was performed with the synthesized cDNA, primers SVE2F 5′ TGGGACGAAGCGGACGATAA 3′ and SVE2R 5′ CTGCTCCGCTTTGGTCGTAT 3′, and Taqman probe 5′ [FAM] CGCATACAGACTTCCGCCCAGT [TAMRA] 3′ (Applied Biosystems) using TaqMan™ Fast Advanced Master Mix (Applied Biosystems, 4444557). Real time PCR was run and analyzed with the 7500 Real Time PCR System (Applied Biosystems). n = 5 for SVNI 3 DPI, n = 4 for CBE + SVNI 3 DPI, n = 10 for SVNI 5 DPI, n = 9 for CBE + SVNI 5 DPI. Levels of viral RNA were calculated based on standard curves and data are presented as plaque forming unit equivalents (pfuE)/μg RNA. Standard curves were generated by serial dilutions of cDNA synthesized from RNA that was extracted from SVNI stock.

### RNA-Seq

#### RNA purification

Brains (n = 3 for each group) were bisected into right and left hemispheres and half were flash-frozen in liquid nitrogen and stored in − 80 °C conditions until use. RNA was isolated using an RNeasy mini kit (Qiagen, Hilden, Germany). RNA quality was examined with the Agilent 2200 TapeStation system (Agilent Technologies); RNA Integrity Number (RIN) values of each sample were determined. Samples of RIN ≥ 8.6 were used for sequencing.

#### Library preparation and sequencing

Libraries were prepared using the INCPM-mRNA-seq protocol. Briefly, the polyA fraction (mRNA) was purified from 500 ng of total input RNA followed by fragmentation and the generation of double-stranded cDNA. After Agencourt Ampure XP beads cleanup (Beckman Coulter), end repair, A base addition, adapter ligation and PCR amplification steps were performed. Libraries were quantified by Qubit (Thermo fisher scientific) and TapeStation (Agilent). Sequencing was done on 1 lane of Hiseq 2500 V4 (Illumina; single read sequencing), allocating ~ 20 M reads per sample.

#### Bioinformatics

Poly-A/T stretches and Illumina adapters were trimmed from the reads using cutadapt; any reads shorter than 30 bp were discarded. Reads were then mapped to the M. musculus GRCm38 reference genome using STAR [17], supplied with gene annotations downloaded from Ensembl (and with EndToEnd option and outFilterMismatchNoverLmax was set to 0.04). Expression levels for each gene were quantified using htseq-count [1] (“HTSeq,” n.d.), using the gtf above. TPM values were estimated independently using Kallisto [5]. Raw *P* values were adjusted for multiple testing using the procedure of Benjamini and Hochberg.

#### Analysis

Expression of mRNA was quantified using DESeq following normalization of library size with Pipeline [41]. Gene lists were created by criteria based on an absolute linear fold change ≥ 2.0, *fdr *≤ 0.05. Enriched pathways in the resulting gene lists were analyzed with IPA (Ingenuity^®^ Systems, www.ingenuity.com) and heat maps were generated using Partek^®^ software (Partek Inc., St. Louis, MI, USA) [42]. Venn diagrams were visualized using BioVenn (http://www.biovenn.nl/). Biological Processes were analyzed with gene ontology (GO) [2, 60, 84].

### Cell enrichment clusters

A list of myeloid lineage cells and astrocytes-enriched gene expression were generated based on previous published cell type specificity expression signatures [33, 93, 94]. Only genes that were found in all three studies were included.

### Quantitative (real-time) RT-PCR

RNA was isolated using an RNeasy mini kit (Qiagen, Hilden, Germany). RNA (1 μg) was reverse-transcribed using the qScript cDNA Synthesis Kit (Quanta, 95047). Real-time PCR was conducted with PerfeCTa SYBR Green FastMix Low ROX (Quanta, 95074) and analyzed with the 7500 Real Time PCR System (Applied Biosystems). The relative amounts of mRNA were calculated from the cycle threshold (Ct) values using HPRT for normalization. (primer sequences, Additional file 1: Table S5).

### Immunofluorescence

Brains (n = 3 for each group) were divided into right and left hemispheres and half were drop-fixed in 4% paraformaldehyde (PFA) overnight, then immersed in 70% ethanol overnight, embedded in paraffin blocks, sectioned to 4 μm, mounted on Superfrost slides and stored at 4 °C until use. Paraffin sections were deparaffinized and antigen retrieval was performed with 10 mM ETDA (pH 8) for 10 min. In order to reduce the non-specific signal, a blocking solution of 20% horse serum (Vector, Burlingame, CA, USA) in 0.5% Triton-X-100 was applied to sections for 90 min. Sections were subsequently incubated with the following antibodies: (1) an anti-IBA1 antibody (1:100, Wako Chemicals, 019-19470), (2) anti-GFAP (1:100, Synaptic Systems, 173 004), and (3) an anti-TMEM119 (1:100, abcam, ab209064) with an anti-GAL3 antibody (1:100 Cedarlane, CL8942AP). Antibodies were diluted into 0.5% Triton-X-100 in PBS containing 2% horse serum overnight at room temperature, and then overnight again at 4 °C. In order to detect TMEM119, sections were incubated with a biotinylated donkey anti-mouse antibody (1:100 Jackson ImmunoResearch, 715-065-151) for 90 min at room temperature, followed by Streptavidin Allophycocyanin (Sa-Apc) (1:100, eBioscience, 7-4317-82) for 1 h. Sections stained against IBA1, were incubated with a donkey anti-rabbit Cy3 antibody (1:100, Jackson ImmunoResearch 711-165-152), while sections stained with an anti-GAL3 antibody and an anti-GFAP antibody were incubated with a donkey anti-rat Cy2 antibody (1:100, Jackson ImmunoResearch, 712-546-153) and a donkey anti-guinea pig Cy3 antibody (1:100, Jackson ImmunoResearch, 706-586-148). With the exception of biotinylated antibodies that were diluted in PBS with 2% horse serum, all other secondary antibodies were diluted in PBS without horse serum and Triton-X-100. Counterstaining was performed with Hoechst (1:1500, 7 min, Molecular Probes, Eugene, OR, USA).

The number of antigen positive puncta were quantified from transverse section of one cerebral hemisphere at the level of 2× using ImageJ software.

### Microglia and monocyte-derived macrophages (Mo-MΦ) isolation

The protocol was conducted as previously described [33]. Mice (*n* = 3–5 for each group) were anesthetized with a combination of ketamine and xylazine (100 and 10 mg/kg, respectively) and were perfused transcardially with PBS. Brains were dissected, coarsely chopped and incubated for 20 min at 37  °C in 1 ml HBSS containing 2% BSA, 1 mg/ml collagenase D (Sigma) and 50 µg/ml DNase1 (Sigma). Next the homogenates were filtered through a 150 μm mesh, washed with cold flow cytometry buffer (2% FCS, 1 mM EDTA in PBS without Ca2+ or Mg2+) and centrifuged at 970 g at 4  °C for 5 min. The cell pellet was resuspended with 3 ml of 40% Percoll and centrifuged again at 970 g, no acceleration and braking, at room temperature for 15 min. The cell pellet was resuspended, passed through an 80 µm mesh, washed with 5 ml flow cytometry buffer and centrifuged at 400gat 4  °C for 5 min, followed by antibody labeling and flow cytometry.

### Flow cytometry

Anti-lymphocyte antigen 6 complex locus G6D Ly6G, (Biolegend, PerCP/Cy5.5-RRID: AB_1877271) antibody was used to detect monocytes and granulocytes; high-Ly6G neutrophils were gated out. Next, anti-CD-45 (Biolegend, FITC-RRID: AB_312973) antibody was used to define monocyte-derived macrophage (Mo-MΦ) and microglia, and an anti-CD11b (Biolegend, APC/Cy7-RRID: AB_830642) antibody was used to distinguish lymphocytes. Finally, microglia were identified by high levels of CD11b, moderate levels of CD45 (CD11b^hi^CD45^int^) while Mo-MΦs were high in both CD11b and CD45 (CD11b^hi^CD45^hi^). Analysis was performed on a Fortessa (BD Biosciences, BD Diva Software) and analyzed with FlowJo software (Treestar).

### Quantification and statistical analysis

Statistical analyses were performed as indicated in the figure legends.

The Kruskal–Wallis test was used for experiments where there is no a priori information regarding the specific distribution from which the data originate. Following this test, post hoc pairwise comparisons were performed using Dunn’s test [76]. The Kruskal–Wallis and Dunn’s tests were conducted in R where using the PMCMRplus package (https://www.r-project.org/index.html) [65]. Two-tailed unpaired *t* test was used otherwise. The threshold for significance was chosen to be α = 0.05, i.e., statistical tests that resulted in *p* values of *p* < 0.05 were considered as indicating significant difference. In the figures, *p* values are indicated by asterisks as follows: *p value < 0.05, **p value < 0.01, ***p value < 0.001, ****p value < 0.0001. The exact value of n, representing the number of mice or biological samples in the each experiment, is indicted in the figure legends.

Flow cytometry data were examined using the Chi square test for equality of proportions followed by calculation for each pair of proportions of Cohen’s h to quantify the magnitude of the effect [13]. The proportions comparison tests were conducted in R using the Hmisc package (https://CRAN.R-project.org/package=Hmisc). Comparisons that resulted in both significant *p* values and large effect sizes (i.e., Cohen’s h > 0.8), were indicated by asterisks. In the figures, *p* values are indicated by asterisks as follows: *p value < 0.05, **p value < 0.01, ***p value < 0.001, ****p value < 0.0001. The exact value of n, representing the number of mice or biological samples in the each experiment, is indicted in the figure legends.

For mouse survival, Kaplan–Meier survival curves were generated and analyzed for statistical significance with GraphPad Prism 6.0 [Log-rank (Mantel–Cox) test (conservative)]. There was no exclusion of data points or mice. No randomization or blinding was used.

### Accession codes

The accession codes for the RNA-seq datasets reported in this paper can be found at GEO: GSE142485 (https://www.ncbi.nlm.nih.gov/geo/query/acc.cgi).

## Results

### Accumulation of GlcCer confers resistant to infection with neuroinvasive Sindbis virus

**Fig. 1:**
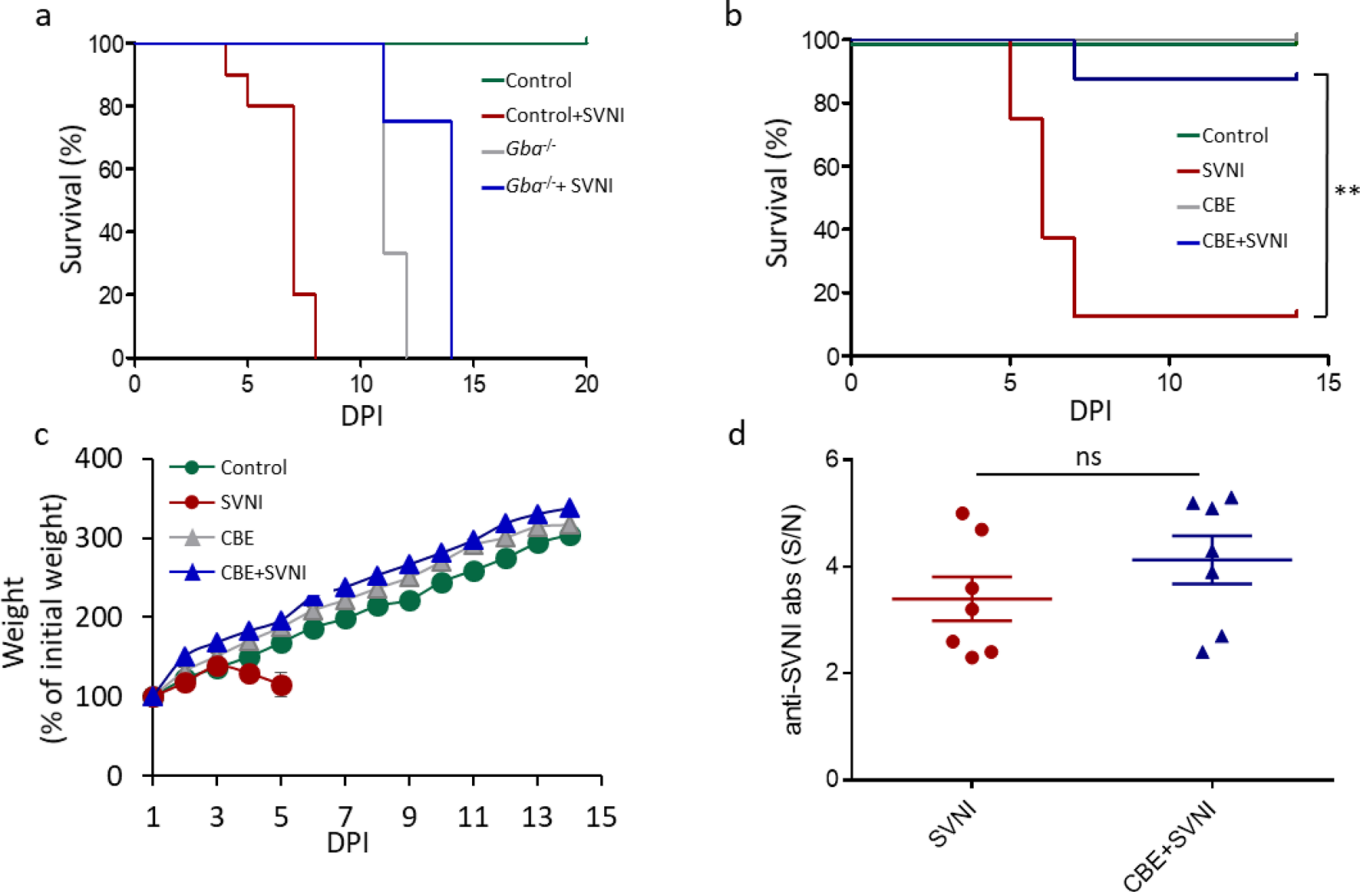
nGD mice are more resistant to SVNI. **a** Survival curve of *Gba*^flox/flox^; nestin-Cre mice (*Gba*^−/−^) and their respective *Gba*^flox/+^; nestin-Cre mice littermate controls (control), uninfected or infected with a lethal dose (5 pfu) of SVNI on 16 day of age. *n* = 4 for *control, n* = 10 for SVNI*, n* = 3 for *Gba*^−/−^, *n* = 4 for *Gba*^−/−^ + SVNI. **b** Survival rate of C57BL/6 mice untreated (control) or treated with CBE (50 mg/kg per day) from 13 day of age, uninfected or infected with a lethal dose (30 pfu) of SVNI on 21 days of age. (n = 8 mice/group). Log-rank test for comparisons of Kaplan–Meier survival curves indicated a significant decrease in the mortality of SVNI + CBE compared to SVNI mice. **p < 0.01. **c** Body weight (% of infection day 0) of C57BL/6 mice untreated (control) or treated with CBE (50 mg/kg per day) from 13 days of age, uninfected or infected with a lethal dose (30 pfu) of SVNI on 21 days of age. Results are mean ± SEM (n = 8 mice/group). In the SVNI group, only 3 mice survived 6 DPI, thus the last point on the graph is 5 DPI. **d** Serum levels of anti-SVNI antibodies in C57BL/6 mice untreated (control) or treated with CBE (50 mg/kg per day) from 13 days of age, uninfected or infected with a lethal dose (30 pfu) of SVNI on 21 days of age. Anti-SVNI antibody levels were measured by ELISA 5–6 DPI. The signal/noise (S/N) ratios of SVNI and CBE + SVNI were determined by dividing the mean absorbance of test sera with the mean absorbance of control and CBE mice, respectively. Results are mean ± SEM (n = 7 for infected groups, n = 3 for uninfected groups). Statistical analysis was performed using a two-tailed unpaired t-test. *ns* not significant

To determine whether GlcCer accumulation in the brain modulates viral pathogenesis, and/or whether viral infection of the CNS affects the severity of nGD pathology, we infected nGD mice with a lethal dose of SVNI. We initially used *Gba*^flox/flox^; nestin-Cre mice in which GCase deficiency is restricted to cells of neuronal lineage [19], thus allowing to specifically study the effect of nGD brain pathology rather than other visceral organs on viral infection. In contrast to control mice, which had a median survival of 7 days post infection (DPI) with SVNI, infected *Gba*^flox/flox^; nestin-Cre mice had a longer lifespan (Fig. 1a) with median survival of 14 days, similar to uninfected *Gba*^flox/flox^; nestin-Cre mice (median of 11 days). Since only 25% of the pups in any given litter are *Gba*^flox/flox^; nestin-Cre, the experiment is limited and performed on a low number of animals. Moreover, the *Gba*^flox/flox^; nestin-Cre mouse is very severe in disease progression, with mortality at 3–4 weeks of age (Fig. 1a) [19], limiting the available window to perform experiments. Thus, we examined another independent, less severe nGD mouse model, namely a chemically-induced model in which an irreversible GCase inhibitor, conduritol B epoxide (CBE) [39], was injected i.p daily into C57BL/6 mice. Injection of mice with CBE induces symptoms typical of neuronal forms of GD [86]. CBE (50 mg/kg/day) administration from 8 days before SVNI infection results in accumulation of GlcCer in the brain and brain inflammation with no overt signs of nGD disease [86]. Similar to the *Gba*^flox/flox^; nestin-Cre mouse, CBE-treated mice were more resistant to SVNI infection (hereafter referred to as CBE + SVNI mice) with undefined median survival compared to 6 days of the control (Fig. 1b).

Notably, whereas control mice infected with SVNI (hereafter referred to as SVNI mice) displayed typical signs of morbidity i.e., weight loss and manifestations of CNS viral infection (Fig. 1c) such as hind limb paralysis, the signs of disease in CBE + SVNI mice were ameliorated (Fig. 1c). No difference was observed between the CBE-treated mice (hereafter referred to as CBE mice) and the CBE + SVNI mice, suggesting that SVNI does not influence nGD disease progression or severity.

To confirm that nGD resistance to SVNI is not due to a difference in viral infection efficacy, levels of viral load (viral RNA, Additional file 1: Figure S1) and anti-SVNI antibodies (total, Fig. 1d) in the serum were measured 3 and 5 DPI, respectively. Both SVNI and CBE + SVNI mice had similar viral load levels in the serum at 3 DPI (Additional file 1: Figure S1), and comparable SVNI antibody titers at 5 DPI (Fig. 1d), indicating similar infection and adaptive immune response in both groups.

To establish whether or not the resistance of nGD mice corresponds specifically to the alphavirus SVNI, we infected CBE-treated mice with the encephalitic flavivirus West Nile virus (WNV). Log-rank test-based analysis of life span data revealed a significant effect of CBE (*P* < 0.01) with a 18.2% elevation of median life span in CBE mice relative to untreated (control) animals (CBE: 11 d; Control: 9 d). Moreover, 25% of WNV-infected mice injected with CBE survived the challenge until the end of the experiment (15 DPI), whereas untreated (control) mice did not survive beyond 9 days post infection (Additional file 1: Figure S2).

### The resistance of nGD to SVNI is accompanied by reduced viral load but similar levels of apoptosis in the brain

**Fig. 2:**
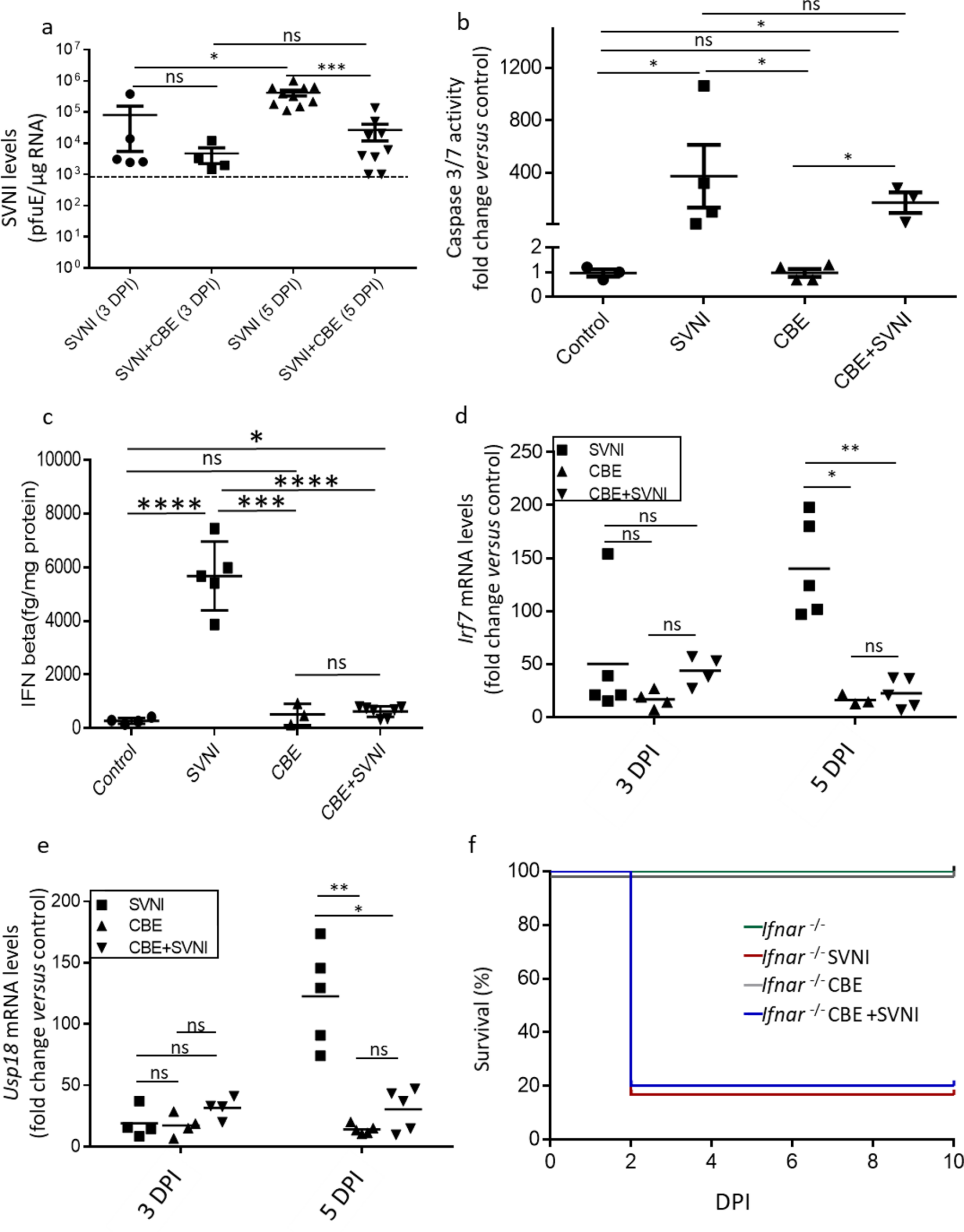
Brain pathology in nGD mice upon SVNI infection. Data were obtained from C57BL/6 mice untreated (control) or treated with CBE (50 mg/kg per day) from 13 days of age, uninfected or infected with a lethal dose (30 pfu) of SVNI at 21 days of age. **a** SVNI viral load in brain homogenates was determined by qPCR at 3 and 5 days post infection (DPI). Levels of viral RNA were calculated based on standard curves and data are presented as plaque forming unit equivalents (pfuE)/μg RNA. Similar levels of viral RNA in the brains of SVNI and CBE + SVNI mice 3 days post SVNI infection. Less viral RNA in CBE + SVNI compared to SVNI mice five-days post SVNI infection. Results are mean ± SEM (n = 5 for SVNI 3 DPI, n = 4 for CBE + SVNI 3 DPI, n = 10 for SVNI 5 DPI, n = 9 for CBE + SVNI 5 DPI). The dotted line reflects the limit of detection. Statistical analysis was performed using a two-tailed unpaired t-test. *p < 0.05, ***p < 0.001, ns, not significant. **b** Activity of caspase-3/7 in brain homogenates 5-DPI. Activities were normalized to 100% of the values of control mice. Values are mean ± SEM. (*n *= 3 for control and CBE + SVNI, n = 4 for SVNI and CBE). *ns* not significant. Statistical analysis was performed using a Kruskal–Wallis test followed by Dunn’s post hoc pairwise comparisons. *p < 0.05. *ns* not significant. **c** ELISA analysis of IFNβ protein levels in cortical homogenates from control, SVNI, CBE, and CBE + SVNI mice 5 DPI. Values are mean ± SEM. (*n *= 4 for control, *n *= 5 for SVNI, *n *= 3 for CBE, *n *= 7 for CBE + SVNI). Statistical analysis was performed using a two-tailed unpaired t-test. *p < 0.05, ****p < 0.0001, *ns* not significant. **d**, **e** qPCR of *Irf7* (**d**) and *Usp18* (**e**) in cortical homogenates from control, SVNI, CBE, and CBE + SVNI mice, 3 and 5 DPI. Results are presented as fold-change *versus* control and are expressed as mean ± SEM. CT values were normalized to levels of HPRT. Statistical analysis was performed using a Kruskal–Wallis test followed by Dunn’s post hoc pairwise comparisons. ***p < 0.001, ****p < 0.0001, *ns* not significant. n = 3–5 for each group. **f** Survival rate of *Ifnar*^–/–^ mice untreated and treated with CBE (50 mg/kg per day) from 13 days of age, uninfected and infected with a lethal dose (5 pfu) of SVNI at 21 days of age. (n = 5–6 mice/group). Log-rank test for comparisons of Kaplan–Meier survival curves indicated no significant difference in the mortality of *Ifnar*^–/–^ +SVNI + CBE compared to *Ifnar*^–/–^ +SVNI mice

To determine whether GlcCer accumulation affects viral entry and replication in the brain, we examined SVNI viral load in the brain by qPCR. While no difference in viral load was observed 3 DPI (the earliest time point at which SVNI was detectable in the brain), a significant decrease in viral RNA levels 5 DPI was observed in CBE mice (Fig. 2a), suggesting that GlcCer accumulation has no effect on virus entry into the brain but rather on viral replication. While neuronal loss in nGD is not apoptotic [91], infection with SVNI leads to high levels of apoptotic cell death [50, 85]. Surprisingly, while no neurological signs were observed in CBE + SVNI mice, there was no difference in levels of the apoptotic markers, caspase 3/7 (Fig. 2b). In addition, TUNEL staining of two mice were in agreement with the shown caspase 3/7 data (data not shown). This suggests that the resistance of CBE + SVNI mice is neither due to neuronal protection nor absence of the virus in the brain.

The role of IFNs as protectors against viral diseases, including SVNI, has been widely studied [57, 74]. Elevation of the type I IFN response in nGD was previously shown [89]. To investigate the possibility that the resistance of CBE + SVNI mice is due to activation of the type I IFN response due to accumulation of GlcCer, we measured levels of IFNβ in the brains of mice in each condition (Fig. 2c). At 5 DPI, levels of IFNβ were much higher in the brains of SVNI mice (5673 ± 572.4 fg/mg protein) compared to SVNI + CBE mice (627.0 ± 74.28 fg/mg protein). However, in the brains of CBE mice, the ELISA was not sensitive enough to detect the elevation of IFNβ compared to control mice, in agreement with previously described data [89]. Thus, to evaluate the activation of the type I-IFN response, we measured levels of *Irf7* and *Usp18* by qPCR. *Irf7* and *Usp18* are IFN-stimulated genes, and are among the 10-most up-regulated genes in the brain of nGD mice [89]. Levels of *Irf7* and *Usp18* were up-regulated at 3 DPI in the brains of SVNI mice (~ 50 ± 26 and ~ 19 ± 6-fold change, respectively), CBE mice (~ 16 ± 4 and 17 ± 4-fold change, respectively) and CBE + SVNI mice (~ 43 ± 7 and 31 ± 4-fold change, respectively) (Fig. 2d, e), indicating similar levels of the type I IFN response in both SVNI and CBE brains upon viral entry (Fig. 2a). At 5-DPI, levels of *Irf7* and *Usp18* were increased to a greater extent in the brains of SVNI mice (~ 140 ± 20 and 122 ± 18-fold change, respectively) compared to 3 DPI. However, no increase was observed in CBE mice (~ 16 ± 3 and 14 ± 2-fold change, respectively) and in CBE + SVNI mice (~ 22 ± 6 and 30 ± 7-fold change, respectively) (Fig. 2d, e), indicating a controlled inflammatory response in the CBE + SVNI mice.

The similar IFN response at 3 DPI in all three groups indicates that the IFN response seen in CBE treated mice on day 3 alone cannot explain the differential suppression of viral pathology, as otherwise it would have also been suppressed in mice infected with SVNI. However, in SVNI mice, the IFN response was induced in response to infection, while in the CBE mice it was induced prior to viral infection, which may be sufficient to render protection. To ascertain the requirement of type I IFN response in mediating the resistance of CBE-treated mice to SVNI-infection, IFN-I receptor-deficient (*Ifnar1*^–/–^) mice [61] were treated with CBE and infected with SVNI. Unlike wild type mice, which are more resistance to SVNI-infection upon treatment with CBE (Fig. 1b), no difference in the lifespans of SVNI-infected *Ifnar1*^–/–^ mice was observed upon CBE injection (Fig. 2f).

In order to shed light on the mechanism governing resistance against virus infection in the nGD brain, we performed high-throughput RNA sequencing (RNAseq) on RNA isolated from half brains of control, SVNI, CBE and CBE + SVNI mice 5 DPI.

**Fig. 3:**
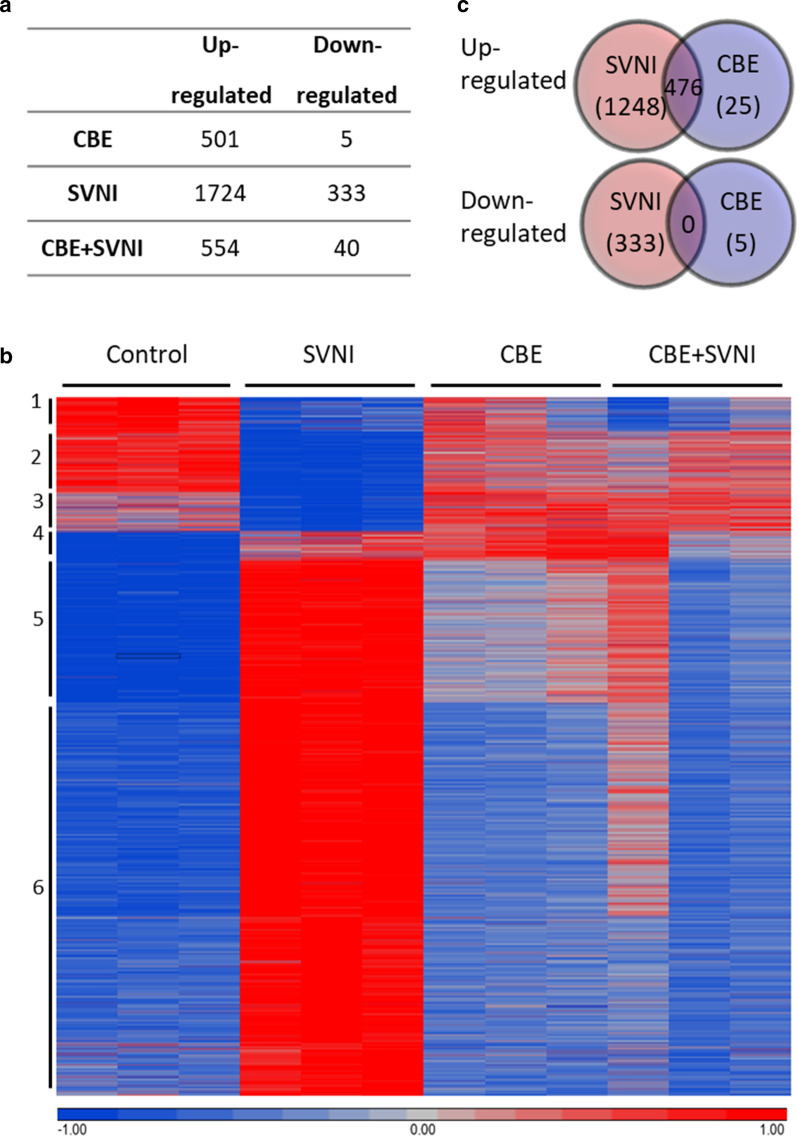
RNAseq shows differences in CNS-immune cell activation between CBE-treated and SVNI-infected mice. **a** Table summarizing the number of differentially expressed genes from RNAseq analysis of the brains of control, SVNI, CBE, and CBE + SVNI mice 5 DPI. The numbers indicate the numbers of genes that were twofold of control, *p* < 0.05. n = 3 for each group. **b** Heat maps of RNAseq data comparing differentially expressed genes in brains of control, SVNI, CBE, and CBE + SVNI mice 5 DPI. A horizontal line shows the expression of a gene in different groups. The expression of such a gene is significantly different in at least one of the pairwise comparisons between different groups. Different colors indicate different levels of gene expression: from red to blue value ranges from high to low, respectively. Clusters numbers are on the left. Data from triplicate samples are shown. Datasets can be found in Additional file 3: Table S2. **c** Venn diagram of differentially-expressed genes in the brains of 5 DPI SVNI and CBE mice. The numbers of shared and unique genes are listed. Overlap was visualized using BioVenn

501 genes were up-regulated in CBE mice *versus* control mice and 5 genes were down-regulated. 1724 genes were up-regulated in SVNI mice *versus* control mice and 333 genes were down-regulated. 554 genes were up-regulated in CBE + SVNI mice *versus* control mice and 40 genes were down-regulated (see Fig. 3a, and Additional file 2: Table S1 for a complete list of the differentially-expressed genes (DEG)) (absolute fold-change ≥ 2, *p* value ≤ 0.05). Gene expression in CBE + SVNI brains was similar to CBE alone [(no statistically significant differentially-expressed transcripts (fold-change ≥ 2, *p* value ≤ 0.05)] rather than the SVNI brains (Fig. 3b, Additional file 2: Table S1), correlating to their resistance against SVNI-induced encephalitis. Up-regulated genes were next subjected to gene ontology (GO) analysis. Among the up-regulated genes, innate immune response pathways were highly enriched in both CBE and SVNI mice (Additional file 4: Table S3). Moreover, a comparison of the enriched biological processes between CBE and SVNI mice indicates numerous corresponding pathways.

To identify pathways in the CBE brains which might be necessary for the viral resistance observed in CBE + SVNI mice, we compared the genes that were differentially-expressed in SVNI *versus* CBE-treated brains (Fig. 3c). Surprisingly, 95% (476 out of 501) of the genes that were up-regulated in the brains of CBE mice were also up-regulated in the brains of SVNI mice. Only 25 genes were uniquely up-regulated in CBE brains, making them considerable candidates for SVNI-resistance genes. Four of the 25 genes did not differ statistically between CBE and SVNI (Table 1). Analysis of the remaining 21 genes indicates that most of them are microglia (MG)- enriched genes (Table 1, Additional file 1: Figure S3). Interestingly, not only were the MG-enriched genes, *Fcrls* and *Gpr34,* up-regulated in the CBE mice, they were also down-regulated in the SVNI mice (fivefold and twofold respectively). qPCR of *Fcrls*, *Gpr34*, *Trem2*, and *Cx3cr1* confirmed the RNAseq results (Additional file 1: Figure S4).

**Table 1:** List of 25 genes up-regulated in the brains of CBE-treated mice but not in SVNI-infected mice

	SVNI versus control	CBE versus control	CBE + SVNI versus control
Statistically significant difference between CBE and SVNI			
*Fcrls*	0.2^¶^ ± 0.0	3.5 ± 0.2	2.4 ± 0.1
*Gpr34*	0.5^¶^ ± 0.1	2.1 ± 0.2	1.5 ± 0.1
*Trem2*	0.7 ± 0.1	5.5 ± 0.4	3.6 ± 0.8
*Cx3cr1*	0.9 ± 0.1	2.5 ± 0.1	2.0 ± 0.3
Ifi27	0.9 ± 0.1	2.6 ± 0.1	2.4 ± 0.3
*Siglech*	1.0 ± 0.3	2.2 ± 0.2	1.9 ± 0.4
Hexa	1.2 ± 0.1	2.1 ± 0.1	1.7 ± 0.2
H2-Ob	1.2 ± 0.4	4.5 ± 0.8	4.3 ± 0.9
*Itgb5*	1.3 ± 0.2	2.3 ± 0.1	1.9 ± 0.3
*Csf1r*	1.3 ± 0.2	2.5 ± 0.1	2.2 ± 0.4
Cybrd1*	1.3 ± 0.4	3.2 ± 0.5	2.4 ± 0.5
Cd33	1.3 ± 0.3	2.9 ± 0.2	2.5 ± 0.6
*Hpgds*	1.3 ± 0.2	2.7 ± 0.3	1.9 ± 0.4
*Ctsd*	1.4 ± 0.1	3.1 ± 0.3	2.2 ± 0.4
Hexb	1.5 ± 0.2	3.2 ± 0.3	2.7 ± 0.6
Spp1	1.5 ± 0.0	2.1 ± 0.3	1.5 ± 0.2
Gusb	1.7 ± 0.1	2.4 ± 0.4	2.0 ± 0.4
Capn3	1.9 ± 0.3	2.6 ± 0.2	2.3 ± 0.3
*H2*-*Oa*	2.0 ± 1.2	5.5 ± 0.2	4.9 ± 1.5
Lpar5	2.1 ± 0.2	3.0 ± 0.2	2.7 ± 0.4
*Lilra5*	2.1 ± 0.1	6.4 ± 1.0	4.5 ± 1.4
Not statistically significant difference between CBE and SVNI			
*Ang*	1.7 ± 0.5	2.4 ± 0.6	1.7 ± 0.6
Abcc3	1.7 ± 0.2	2.3 ± 0.4	2.1 ± 0.4
Lpcat2	1.9 ± 0.2	2.1 ± 0.1	1.9 ± 0.4
Arhgap19	1.8 ± 0.2	2.1 ± 0.3	1.8 ± 0.3

RNAseq analysis of brains of control, SVNI, CBE, and CBE + SVNI mice 5 DPI. The numbers indicates fold-change versus control

^¶^Significantly down-regulated (≥ 2.0, *fdr *≤ 0.05). Genes in bold font indicate higher expression levels in microglia than all other non-myeloid cells in the CNS. Italicized genes also indicate enrichment in microglia over other myeloid cells

*Expression at or more highly in astrocytes than other cells in the CNS

In addition to MG-enriched genes, the astrocyte-enriched gene *Cybrd1* [14] was also up-regulated in CBE but not in SVNI (Table 1). These data suggest that CBE and SVNI brains diverge in activation of the two major inflammation-driving endogenous CNS cells, MG and astrocytes.

### Increased activation of myeloid lineage cells and altered astrocyte activation in SVNI versus CBE mice

To assess the activation state of myeloid lineage cells and astrocytes in the brain, we performed immunohistochemistry on 5 DPI cortical sections with IBA1 and GFAP. In addition, we generated a list of genes enriched in myeloid lineage cells and astrocytes based on published cell type specificity expression signatures [33, 93, 94] and examined their expression by RNAseq.

**Fig. 4:**
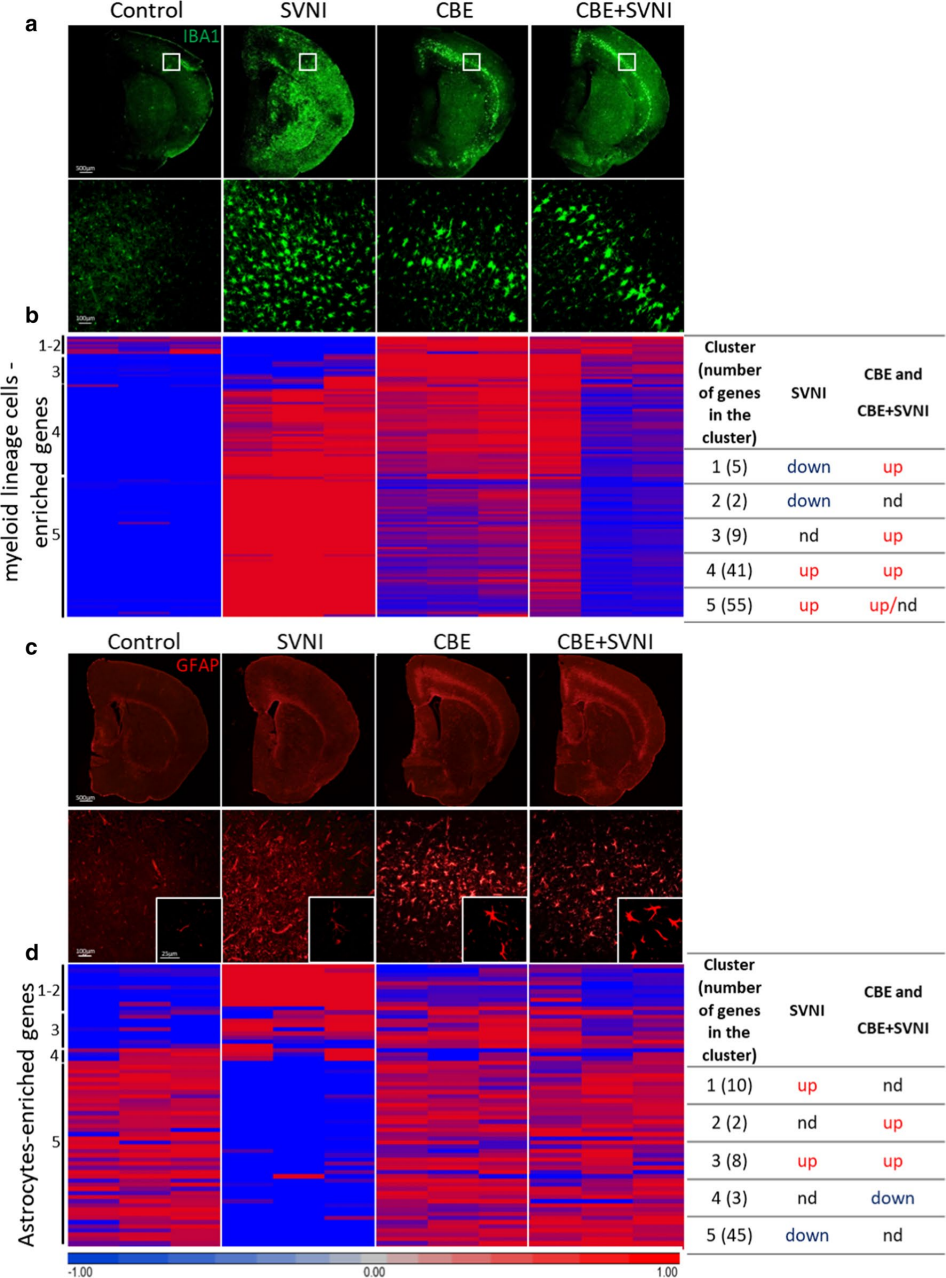
Activation of myeloid lineage cells with abrogated activation of astrocytes upon infection with SVNI. Data were obtained from C57BL/6 mice untreated (control) or treated with CBE (50 mg/kg per day) from 13 days of age, uninfected or infected with a lethal dose (30 pfu) of SVNI on 21 days of age. Data were obtained 5–6 DPI. **a** Immunofluorescence using an anti-IBA1 (green) antibody (×2 magnification; upper panel, ×20 magnification; lower panel). IBA1 staining of ×2 magnification was evaluated using ImageJ. Control, SVNI, CBE, and CBE + SVNI contain ~ 382 ± 122, 1801 ± 144, 987 ± 177, and 1157 ± 182 IBA1-positive cells, respectively. Results are representative of three biological replicates. **b** Heat maps of RNAseq comparing brains of control, SVNI, CBE, and CBE + SVNI mice, represented by lists of 112 genes enriched in myeloid lineage cells showing enrichment and de-enrichment of mRNAs in different samples. A summary of the clusters is presented in the table (right). Datasets in Datasets in Additional file 5: Table S4. Each column represents an individual mouse, n = 3 for each group. nd no difference. **c** Immunofluorescence using an anti-GFAP (red) antibody (×2 magnification; upper panel, ×20 magnification; lower panel). GFAP staining of ×2 magnification was evaluated using ImageJ. Control, SVNI, CBE, and CBE + SVNI contain ~ 666 ± 48, 823 ± 167, 990 ± 185, and 1131 ± 44 GFAP-positive cells, respectively. Results are representative of three biological replicates. **d** Heat maps of RNAseq from brains of control, SVNI, CBE, and CBE + SVNI mice, represented by lists of 68 genes enriched in astrocytes showing enrichment and de-enrichment of mRNAs in the different samples. A summary of the clusters is presented in the table (right). Datasets in Additional file 5: Table S4. Each column represents an individual mouse, n = 3 for each group

Profound activation of MG/macrophages (MΦ) (IBA1-positive) was detected in SVNI brains (~ 382 ± 122, 1801 ± 144, 987 ± 177, and 1157 ± 182 IBA1-positive cells, in control, SVNI, CBE, CBE + SVNI brain sections, respectively) (Fig. 4a). Whereas activation of MG/MΦ was restricted to defined brain areas such as layer V of the cortex [22] in CBE brains, the activation of MG/MΦ is spread throughout the entire brain upon SVNI infection (Fig. 4a). Corresponding to the extensive elevation in detectable MG/MΦ, a significant elevation in myeloid lineage cell-enriched genes was observed in SVNI brains (Fig. 4b, clusters 4 and 5, Additional file 5: Table S4). Elevation of myeloid lineage cell-enriched genes was also observed in CBE brains but to a lesser extent when compared with that of SVNI brains (Fig. 4b, Additional file 5: Table S4).

Activation of microglia and astrocytes is a common feature of neurodegenerative disorders [32] and correlates with neuronal loss and disease severity in nGD [22]. Astrocyte activation (GFAP-positive) in CBE mice was detected in the same brain area as MG/MΦ activation (Fig. 4a, c). However, while massive MG/MΦ activation was detected in the SVNI brains (Fig. 4a), reactive astrocytes were scarcely detectable (~ 666 ± 48, 823 ± 167, 990 ± 185, and 1131 ± 44 GFAP-positive cells, in control, SVNI, CBE, CBE + SVNI brain sections, respectively) (Fig. 4c). Reactive astrocytes in the CBE brain exhibit a striking intensification in GFAP immunoreactivity and appeared hypertrophic (Fig. 4c), while the astrocytes in the SVNI brains showed a lesser increase in GFAP immunoreactivity and are not hypertrophic (Fig. 4c). Moreover, most of the astrocyte-enriched genes were down-regulated in the SVNI brain (Fig. 4d, cluster 5, Additional file 5: Table S4). The CBE + SVNI brain demonstrates a similar pattern to the CBE brain in terms of MG/MΦ and astrocytic activation (Fig. 4a–d). Together, these data indicate differential activation states of astrocytes between nGD and SVNI-infection conditions.

### Differential activation of microglia by GlcCer accumulation and SVNI

IBA1 staining demonstrated activation of myeloid cells in both CBE and SVNI mice. However, the difference in MG activation between CBE and SVNI mice, as indicated by RNAseq, cannot be explained by IBA1 staining. Myeloid cell populations in the CNS during neuropathology are composed of endogenous microglia, non-parenchymal macrophages, and monocyte-derived macrophages (Mo‐MΦ), all of which can be detected with IBA1 [26, 51, 67].

**Fig. 5:**
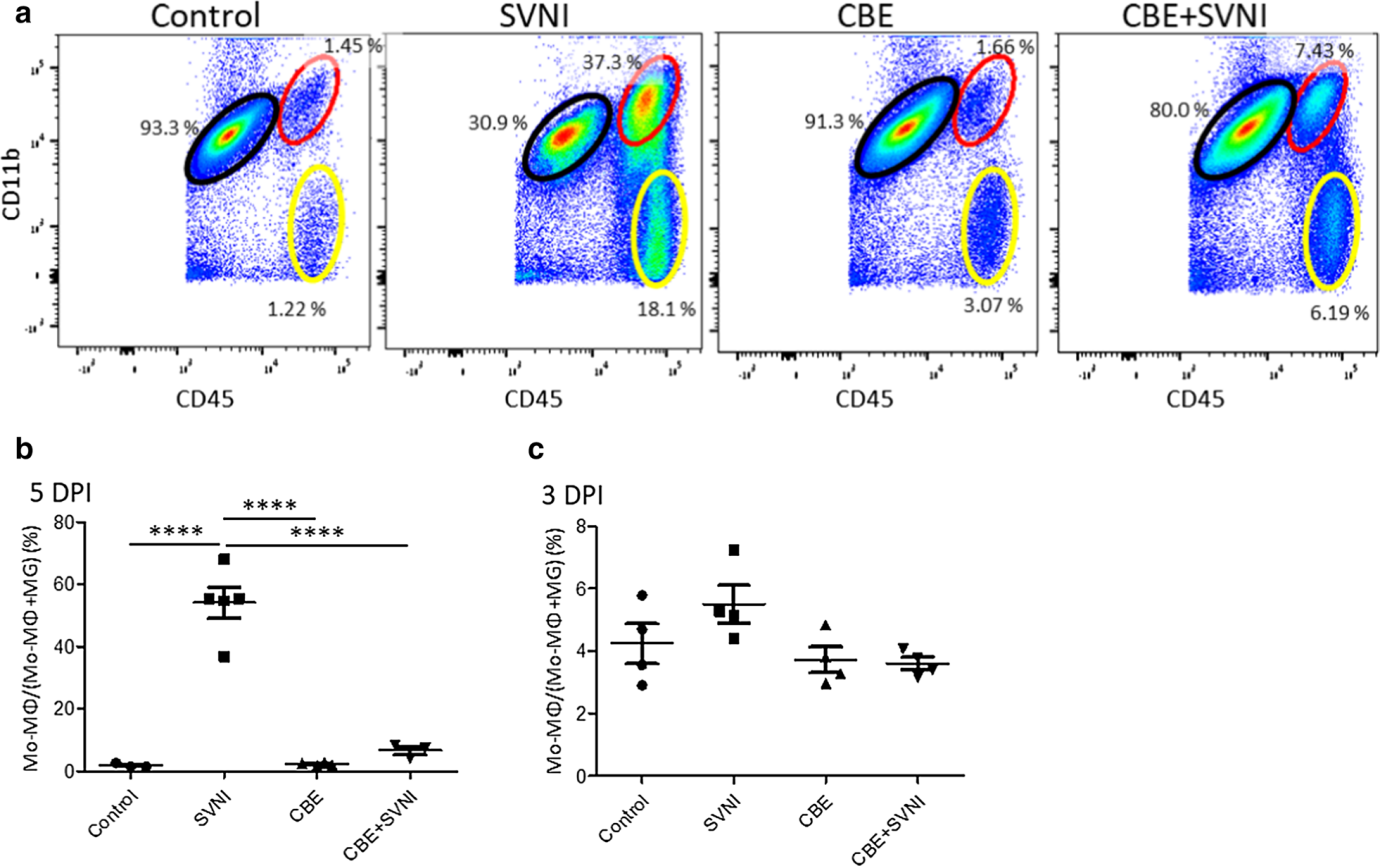
High levels of MΦ infiltration are observed in the brains of SVNI-infected mice. Data were obtained from C57BL/6 mice untreated (control) or treated with CBE (50 mg/kg per day) from 13 day of age, uninfected or infected with a lethal dose (30 pfu, 3LD50) of SVNI on 21 day of age. **a** Flow cytometry analysis of myeloid cells in the CNS of control, SVNI, CBE, and CBE + SVNI mice 5-DPI. Microglia express high levels of CD11b and intermediate levels of CD45 (indicated by the *black* ellipse), whereas monocyte-derived macrophage (Mo-MΦ) express high levels of both CD11b and CD45 (indicated by the *red* ellipse). Lymphocytes express high levels of CD45 and are negative for CD11b ((indicated by the *yellow* ellipse). *n* = 3–5 for each group. **b**, **c** The percentage of Mo-MΦ out of total myeloid lineage cells [Mo-MΦ + microglia (MG)] in the brain of control, SVNI, CBE, and CBE + SVNI mice 5- days (**b**) or 3-days (**c**) post infection. Pairs of proportions that resulted in both significant *p* values and large effect sizes (i.e., Cohen’s h > 0.8) were indicated according to their *p* values: *p < 0.05, ***p < 0.001, ****p < 0.0001

**Fig. 6:**
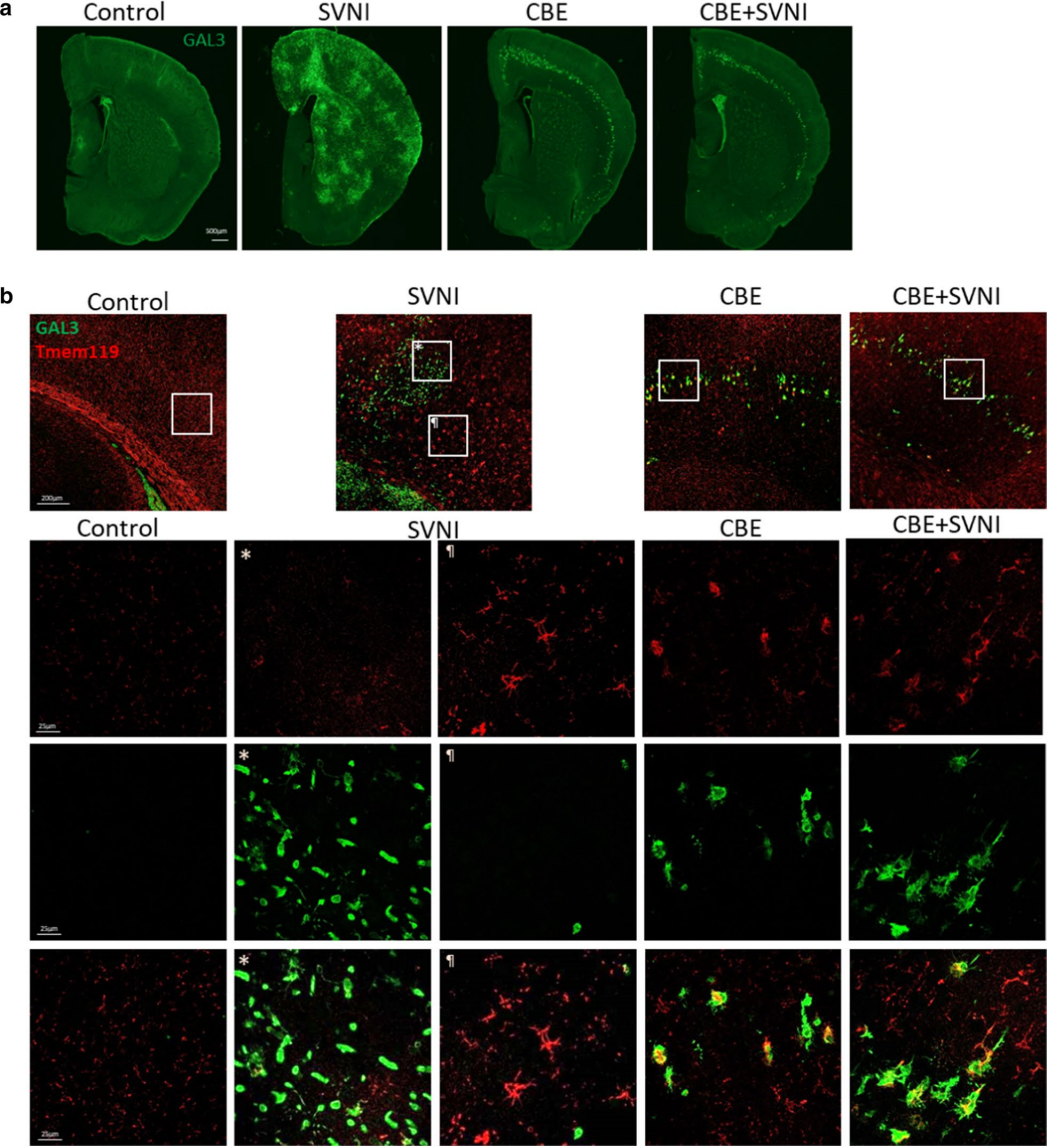
Distinct patterns of microglia activation in brains of SVNI-infected versus nGD mice. Data were obtained from C57BL/6 mice untreated (control) or treated with CBE (50 mg/kg per day) from 13 day of age, uninfected or infected with a lethal dose (30 pfu, 3LD50) of SVNI on 21 day of age. **a** Immunofluorescence of brains of control, SVNI, CBE, and CBE + SVNI mice using anti-GAL3 (green) antibody. GAL3 staining was evaluated using ImageJ. Control, SVNI, CBE, and CBE + SVNI contain ~ 308 ± 16, 1548 ± 644, 663 ± 46, and 713 ± 92 GAL3-positive cells, respectively. Results are representative of three biological replicates. **b** Double immunofluorescence of brains of control, SVNI, CBE, and CBE + SVNI mice using anti-GAL3 (green) and anti-Tmem119 (red) antibodies. ×2 (upper panel) and ×20 (lower panel) magnification images. Data were obtained 5-DPI. Results are representative of three biological replicates

Flow cytometry was next used to distinguish between Mo-MΦs and resident microglia. Levels of Mo‐MΦs (CD45^hi^CD11b^hi^) and microglia (CD45^low^CD11b^hi^) were measured in the brains of control, CBE, SVNI and CBE + SVNI mice, 3 and 5-DPI (Fig. 5a–c). As recently shown [12], CBE mice displayed basal levels of Mo-MΦs (2.2 ± 0.2% of combined microglia and macrophages populations), similar to control mice (1.9 ± 0.4%) (Fig. 5a, b). However, SVNI mice exhibited high levels of Mo-MΦ infiltration into the brain (54 ± 5%) 5-DPI (Fig. 5a, b). It should be noted that a small fraction of the CD45^hi^CD11b^hi^ in the SVNI mice may be activated microglia which can increase their CD45 expression upon activation [10]. CBE-treatment results in a significant reduction in the frequency of Mo-MΦ infiltration into the brain upon SVNI-infection (6.7 ± 1.4%). No significant difference was observed between the groups at 3 DPI (Fig. 5c). Similarly, CBE treatment alleviated the level of lymphocyte infiltration upon SVNI infection (Fig. 5a). The flow cytometry analysis indicates a difference in the myeloid cell population in SVNI-induced encephalitis compared to that of nGD. While the nGD brain’s myeloid cells are resident MG with no recruitment of monocytes, massive monocyte infiltration of the brain characterizes the pathological state upon SVNI-infection. Significantly lower levels of monocyte infiltration into CBE + SVNI brains (Fig. 5a, b) correlates to resistance against SVNI infection, suggesting a detrimental role of Mo-MΦs in SVNI pathogenesis. To determine differences in MG activation states between the groups, brains were co-stained with Gal-3 (also known as Mac-2 or LGALS3) and TMEM119. GAL3 is expressed in macrophages and microglia and is a key player in microglial activation [4, 7, 35, 36, 53]. Up-regulation of GAL3 is observed in activated MG during nGD and the marker has been used to identify activated MG in nGD mice [19, 22, 89]. TMEM119 was recently recognized as a specific marker of microglia cells [3], allowing to distinguish between MG and Mo-MΦs. Increased levels of GAL3 were observed in the brains of both SVNI and CBE mice (Fig. 6a). In CBE mice, increased GAL3 was observed in distinct pathological areas as previously described [22], whereas in SVNI mice, GAL3 immunoreactivity was observed throughout the brain (~ 308 ± 16, 1548 ± 644, 663 ± 46, and 713 ± 92 GAL3-positive cells, in control, SVNI, CBE, CBE + SVNI contains, respectively) (Fig. 6a). However, while GAL3 expression was elevated in MG cells (TMEM119-positive) in CBE mice, it was not expressed in MG in the SVNI mice, but rather in non-MG cells (Fig. 6b), indicating dissimilar microglial activation states between SVNI and CBE mice. The MG activation state of CBE + SVNI mice is similar to that of CBE (GAL3-positive), suggesting a beneficial role of GAL3-positive MG in CBE + SVNI mice. Characterization of the GAL3-positive cell populations in SVNI mice remains to be elucidated.

## Discussion

In this study we identified two distinct innate immune responses in the brain: one is activated in response to a chronic intrinsic stimulus (i.e. GlcCer accumulation) and the other activated by an acute extrinsic stimulus (i.e. SVNI infection). Moreover, we demonstrate that the immune response in the brain upon GlcCer accumulation is sufficient to arrest viral spread and protect mice from viral infection of the CNS.

Our results suggest that the resistance of nGD mice to SVNI is mediated by brain pathology arising from GlcCer accumulation and not due to activation of pathways in peripheral organs. We reason such in a twofold manner: (1) the *Gba*^flox/flox^; nestin-Cre mouse, in which GD pathology is restricted to the CNS, demonstrated resistance to SVNI (Fig. 1a); (2) no difference in viral entry of brain was observed, as indicated by similar viral loads in the brains of SVNI and CBE + SVNI mice 3 DPI (Fig. 2a), together with comparable levels of apoptotic markers (Fig. 2b). The similarity of apoptotic cell quantities suggests that the resistance of nGD mice is not explained by protection against apoptosis, and that levels of cell death in the brain are not correlated with disease severity, as previously shown [29, 62]. Moreover, when CBE treatment was begun only 2 days prior to infection (data not shown) rather than 8 days beforehand, the disease outcome was similar in both SVNI and CBE + SVNI mice. Thus, CBE itself does not render viral resistance; it likely initiates one or more down-stream signaling cascades that ultimately protect against viral infection.

In contrast to apoptotic markers, inflammatory markers correlate with disease severity. SVNI mice present the most severe signs and the highest levels of inflammatory markers as indicated by a significant elevation of IBA1-positive cells (Fig. 4a) and up-regulation of inflammatory pathways compared to CBE mice (Additional file 2: Table S1 and Additional file 4: Table S3).

Neuropathology in nGD brain is characterized by altered lysosomes, neuronal loss, and various up-regulated pathways, all of which can potentially confer resistance against viral infection [86–88, 95]. However, our RNAseq analysis revealed fundamental differences between CBE and SVNI mice in genes enriched in innate-immune cells. This result suggests that the prevailing mechanism of resistance may be the innate immune response. Unexpectedly, the majority (95%) of up-regulated genes in the CBE mice were also up-regulated in SVNI mice, despite the fact that the two diseases arise from distinct etiologies (Fig. 3c). Strikingly, most of the genes that were up-regulated only in CBE mice are associated with the innate immune response (Table 1, Additional file 1: Figure S3). Among these genes is the interferon stimulated gene, Interferon alpha inducible protein 27 (*Ifi27*). Interferon alpha-inducible protein 27 like 2A (*Ifi27l2a*) contributes to the innate immune restriction of a flavivirus (west-nile virus [WNV]  [54]), and a coronavirus (murine hepatitis virus [MHV]) but not of an alphavirus (Venezuelan equine encephalitis virus [VEEV]) [11] in mice. Thus, it would be worthwhile to explore the antiviral role of *Ifi27* in alphavirus infections.

The major finding of the RNAseq analysis demonstrates that the main difference between the immune responses to GlcCer accumulation and SVNI-infection is up-regulation of a distinct subset of MG-enriched genes in CBE mice (Table 1, Additional file 1: Figure S3). This is somewhat surprising given the immune response in the SVNI brain is very robust, and yet there are specific MG-enriched genes which are not up-regulated. Interestingly, not only were the MG-enriched genes, *Fcrls* and *Gpr34,* not up-regulated in SVNI mice, they were actually down-regulated (Table 1, Additional file 1: Figure S4). Down-regulation of *Fcrls* and *Gpr34* in MG is associated with aging and genetic diseases, and a role for these genes in MG homeostatic function has been suggested [8, 43]. However, since the RNAseq in this study was performed on half brain tissue rather than isolated MG, single cell transcriptome analysis is required to confirm that the down-regulation of these genes upon SVNI infection is indeed unique to MG. The specific down-regulation of these key inflammatory genes in SVNI mice might imply a mechanism by which the virus modulates expression of these genes and/or suppresses MG-activation in order to evade the immune response. Interestingly, comparison between our RNAseq data and RNAseq obtained from brains of mice upon infection with WNV [47] reveals that all of the genes up-regulated in CBE only (Table 1) were not up-regulated in WNV, similar to the SVNI condition. Moreover, *Fcrls* and *Trem2* were down-regulated in the WNV-infected brain. In addition, CBE-treated mice were more resistant to infection with WNV (Additional file 1: Figure S2), which may imply a broad mechanism by which CBE confers viral-resistance.

The RNAseq analysis points to distinctive activation states of astrocytes and MG in the CBE brain as compared to the SVNI brain (Table 1, Additional file 2: Figures S3, 4b and 4d). Indeed, immunostaining and flow cytometry revealed almost opposite inflammatory responses: while the inflammatory response in CBE brains consisted of reactive astrocytes and MG with no infiltration of Mo‐MΦs, the inflammatory response in SVNI brains revealed massive infiltration of Mo‐MΦs, with altered activation of the CNS resident immune system (Figs. 4, 5). In contrast to the GAL3-positive MG in CBE brains, SVNI brains demonstrated GAL3-negative MG (Fig. 6), suggesting a differential activation state of MG between these two conditions. Genetic neurodegenerative diseases are accompanied by activation of both MG and astrocytes [9, 34, 68, 90]. Perhaps most surprising is the distinct activation state of astrocytes in the SVNI brain, which is interrupted despite a massive inflammatory response (Fig. 4c, d). Activation of astrocytes in response to SVNI infection was shown in culture [6], and an in vivo study demonstrated an increase in *Gfap* promoter activity in SVNI GFAP-luciferase mice [29]. While *Gfap* mRNA levels were up-regulated in SVNI mice 5 DPI (RNA-seq data, Additional file 2: Table S1, Additional file 5: Table S4), GFAP immunofluorescence reveals that this elevation is not accompanied by the classic morphological changes that characterize astrocyte activation (Fig. 4d).

More recently, the interaction between reactive astrocytes, activated microglia, and invading monocytes has been studied, albeit in non-viral models. Secretion of IL-1α, TNF, and C1q by MG is necessary and sufficient to activate astrocytes [52]. Indeed, mRNA levels of all the above-mentioned cytokines were up-regulated in the SVNI brain (Additional file 2: Table S1); however, astrocyte activation appeared to be interrupted or altered (Fig. 4c, d). A stab injury mouse model demonstrated that quantities of monocytes were inversely effected by the presence of reactive astrocytes. Conversely, reducing infiltration of monocytes lead to a strong increase in astrocyte proliferation, revealing a negative-feedback regulation loop between infiltrating monocytes and astrocytes [23]. These studies align with our observation of concurrently high levels of infiltrating monocytes (Fig. 5a, b) and aberrant activation of astrocytes in SVNI brains (Fig. 4c, d). Worthy of consideration is whether or not SVNI and other viruses are capable of directly modifying the activation state of astrocytes and thereby enabling monocyte infiltration, or vice versa, if they induce monocyte infiltration which affects the ability of astrocytes to fully respond to a challenge.

Massive infiltration of Mo-MΦs into the brain upon SVNI infection was significantly reduced in CBE + SVNI mice, correlating closely with their enhanced survival (Fig. 5a, b). Thus, our data suggest that infiltration of Mo-MΦs is not required for an efficient anti-viral immune response, and that infiltrating Mo-MΦs may even be deleterious to the host. Our data are in line with a previous study showing that AMPA receptor antagonists protect from SVNI infection by suppressing mononuclear cell infiltration [29]. Whether the enhanced resistance of CBE mice to SVNI-infection is due to a reduction in Mo‐MΦs infiltration, or due instead to the specific activation state of astrocytes and/or MGs, needs to be further elucidated. Understanding the mechanism by which the innate immune response in nGD protects mice from viral infection may open novel avenues for anti-viral therapy. Interestingly, the blood–brain barrier in nGD is permeabilized [88] even though there is no infiltration of Mo-MΦs into the brain, suggesting an active mechanism in nGD that prevents their recruitment following viral infection of the CNS.

Clearance of SVNI from the CNS was shown to be mediated by antibodies and not by cellular immunity [49]. This is consistent with our data showing similar levels of anti-SVNI antibodies in both SVNI and CBE + SVNI mice (Fig. 1d).

We showed that the levels of both virus and the type I IFN response in SVNI mice are upregulated at 5 DPI compared to 3 DPI, whereas levels in CBE and CBE + SVNI mice were unchanged from 3 DPI to 5 DPI (Fig. 2a, d). Although activation of the type I IFN response in CBE + SVNI mice would be the simplest explanation for their survival, we have shown that both virus and type I IFN levels are similar in both CBE and SVNI brains at 3 DPI, indicating that basal IFN levels in CBE mice cannot explain the divergence in viral pathology and disease severity between them. However, we cannot rule out that activation of IFN prior to viral entry is required for resistance, or distinguish whether different cell types in the brain secrete IFN in CBE *versus* SVNI mice. Our results are consistent with the widely-recognized significance of type I IFNs in mediating host defense against viruses [59]. *Ifnar1*^–/–^ mice are highly susceptible to numerous viruses, including SVNI, resulting in higher viral organ titers and reduced lifespan compared to wild-type mice under the same conditions [57, 74]. Thus, it is not surprising that activation of type I IFN response upon CBE-treatment is necessary to render resistance against SVNI. Indeed, the direct or indirect mechanism of type I IFN response in mediating the viral resistance of CBE + SVNI mice remains to be elucidated. However, we cannot exclude the possibility that *Ifnar1*^–/–^ mice infected with SVNI succumb to systemic disease, rather than a disease of the CNS, and therefore, amelioration of neurological disease by CBE may not be sufficient to alleviate disease symptoms in the case of the *Ifnar1*^–/–^ mouse. It would be intriguing to examine whether genetic diseases with no induction of the type I IFN response, yet characterized by reactive MG and astrocytes, would render more resistance to SVNI-infection.

While activation of the inflammatory response, particularly of type I IFNs, is critical for protection of the host against infectious diseases, a dysregulated host response to infection is detrimental. Several lines of evidence suggest that fatal SVNI encephalitis is mediated by the immune response to viral infection rather than the infection itself per se [29]. Our RNAseq data revealed that the inflammatory response to SVNI infection is more robust compared to that of CBE mice; 1248 genes were uniquely up-regulated in the SVNI mice and the overall fold-change of inflammatory genes was far higher than that of CBE mice. These 1248 up-regulated genes may include those that facilitate deleterious inflammation and ultimately cause mortality. Specifically, pathways related to T cell activation (Additional file 4: Table S3) were among those exclusively upregulated in SVNI mice. This finding is in keeping with previous studies suggesting a role for pathogenic Th17 cells, CD4+, and CD8 + T cells in fatal SVNI encephalitis [45, 46, 73]. Thus, the protection observed in CBE mice could directly result from reducing cytotoxic and harmful pathways that are activated by SVNI, and not merely due to elevation of beneficial pathways. Delineating the mechanism by which CBE mice are resistant to SVNI may enable new strategy for therapeutic intervention.

Activation of immune response in the brain is a hallmark of many neurodegenerative disorders. However, distinctive immune response pathways are activated in response to different stimuli. Even in closely related diseases such as LSDs, distinct inflammatory pathways are activated [89, 90]. Thus, the overall similarity in the immune responses following both GlcCer accumulation and SVNI-infection is highly unexpected. Most of the up-regulated pathways in CBE mice were also up-regulated in SVNI mice, raising the question as to whether GlcCer itself plays a role in SVNI-infection.

The observation that the nGD murine model is more resistant to viral infection leads us to contemplate whether this resistance can bestow any adaptive physiological advantage upon nGD patients. Indeed, it is difficult to argue that nGD-mediated resistance to viruses is advantageous to the host without considering the weight of devastating nGD symptoms in the life of a patient. Nevertheless, whether or not non-neuronopathic GD patients and/or *Gba* carriers are more resistant to viral infections needs to be further clarified and may provide a basis for future studies to bring to light the role of *Gba* mutations.

## Supplementary information

**Additional file 1: Fig. S1**. Similar SVNI viral load in the serum of SVNI and SVNI + CBE mice 3 DPI. SVNI viral load in serum was determined by qRT-PCR at 3 days post infection (DPI). Levels of viral RNA were calculated based on standard curves and data are presented as plaque forming unit equivalents (pfuE)/μg RNA. Similar levels of viral RNA in the serum of SVNI and CBE + SVNI mice 3 days post SVNI infection were detected. Results are mean ± SEM (n = 4). Statistical analysis was performed using two-tailed unpaired t-test. *ns* not significant. **Fig. S2**. nGD mice are more resistance to WNV. Survival rates of C57BL/6 mice untreated (control) or treated with CBE (50 mg/kg per day) from 13 days of age, uninfected or infected with a lethal dose (10 pfu, 3LD50) of WNV on 21 day of age (n = 8 mice/group). Log-rank test for comparison of Kaplan–Meier survival curves indicated a significant decrease in the mortality of CBE + WNV mice compared to WNV-infected animals. **p < 0.01. **Fig S3**. Most genes that were up-regulated in the CBE-only mice are enriched in MG. Expression patterns of the 25 genes up-regulated in CBE-only mice are shown along the cell-type taxonomy. Each row represents one gene, and columns represent cell clusters. MG-cells (Immune) are shown in a box. Colors are proportional to the levels of transcription. The genes were analyzed with http://mousebrain.org/genesearch.html [93]. **Fig. S4**. Validation of RNA-seq data by qPCR. qPCR analysis of *Fcrls*, *Gpr34*, *Trem2*, and *Cx3cr* in cortical homogenates from control, SVNI, CBE, and CBE + SVNI mice 5–6 DPI. Results are presented as arbitrary units (AU) and are expressed as the mean ± SEM. CT values were normalized to levels of HPRT. Statistical analysis was performed using a two-tailed unpaired t test. *p < 0.05, **p < 0.01, ***p < 0.001. n = 4–6 for each group. **Table S5**. Primers used for polymerase chain reaction.

**Additional file 2: Table S1**. Complete list of the differentially-expressed genes (DEG). RNA-seq data obtained from brains of control, SVNI, CBE, and CBE + SVNI mice 5 DPI. Gene lists were created by criteria based on an absolute linear fold change ≥ 2.0, *fdr* ≤ 0.05 .

**Additional file 3: Table S2**. Gene clusters of differentially-expressed genes (DEG). Values that were used for generation of heat maps of RNA-seq data. The heat maps comparing mRNA obtained from brains of control, SVNI, CBE, and CBE + SVNI mice are represented by lists of 2293 genes that were significantly differentially-expressed (fold change ≥ 2.0, *p* ≤ 0.05) in at least one of the groups versus control.

**Additional file 4: Table S3**. List of significantly enriched GO-Slim Biological Process. Biological processes which were up-regulated in both CBE-treated and SVNI-infected brains are highlighted in yellow.

**Additional file 5: Table S4**. Gene clusters of myeloid lineage cells and astrocytes-enriched genes. Values that were used for generation of heat maps of RNA-seq data. The heat maps comparing mRNA obtained from brains of control, SVNI, CBE, and CBE + SVNI mice are represented by lists of 68 and 112 genes enriched in astrocytes and myeloid lineage cells, respectively.
