# Supplementary material for: Innate immune response in neuronopathic forms of Gaucher disease confers resistance against viral-induced encephalitis
Source: Acta Neuropathol Commun. 2020 Aug 24;8:144. doi: 10.1186/s40478-020-01020-6 (PMC7443817; doi:10.1186/s40478-020-01020-6)
Supplement: Supplementary file 1 — Additional file 1: Fig. S1. Similar SVNI viral load in the serum of SVNI and SVNI + CBE mice 3 DPI. SVNI viral load in serum was determined by qRT-PCR at 3 days post infection (DPI). Levels of viral RNA were calculated based on standard curves and data are presented as plaque forming unit equivalents (pfuE)/μg RNA. Similar levels of viral RNA in the serum of SVNI and CBE + SVNI mice 3 days post SVNI infection were detected. Results are mean ± SEM (n = 4). Statistical analysis was performed using two-tailed unpaired t-test. ns not significant. Fig. S2. nGD mice are more resistance to WNV. Survival rates of C57BL/6 mice untreated (control) or treated with CBE (50 mg/kg per day) from 13 days of age, uninfected or infected with a lethal dose (10 pfu, 3LD50) of WNV on 21 day of age (n = 8 mice/group). Log-rank test for comparison of Kaplan–Meier survival curves indicated a significant decrease in the mortality of CBE + WNV mice compared to WNV-infected animals. **p < 0.01. Fig S3. Most genes that were up-regulated in the CBE-only mice are enriched in MG. Expression patterns of the 25 genes up-regulated in CBE-only mice are shown along the cell-type taxonomy. Each row represents one gene, and columns represent cell clusters. MG-cells (Immune) are shown in a box. Colors are proportional to the levels of transcription. The genes were analyzed with http://mousebrain.org/genesearch.html [93]. Fig. S4. Validation of RNA-seq data by qPCR. qPCR analysis of Fcrls, Gpr34, Trem2, and Cx3cr in cortical homogenates from control, SVNI, CBE, and CBE + SVNI mice 5–6 DPI. Results are presented as arbitrary units (AU) and are expressed as the mean ± SEM. CT values were normalized to levels of HPRT. Statistical analysis was performed using a two-tailed unpaired t test. *p < 0.05, **p < 0.01, ***p < 0.001. n = 4–6 for each group. Table S5. Primers used for polymerase chain reaction. [file 40478_2020_1020_MOESM1_ESM.docx]

**Supplemental Figures**

Fig S1.


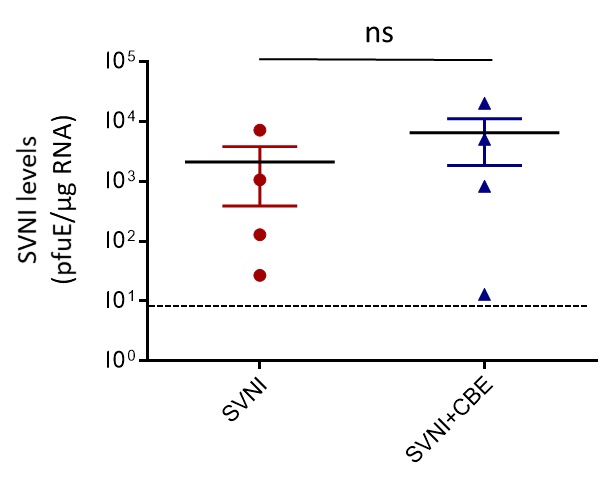


Fig S2.

*
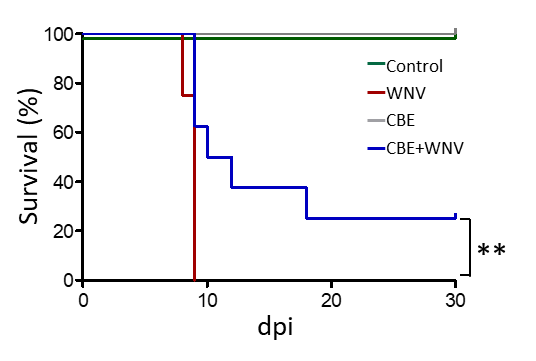
*

Fig. S3


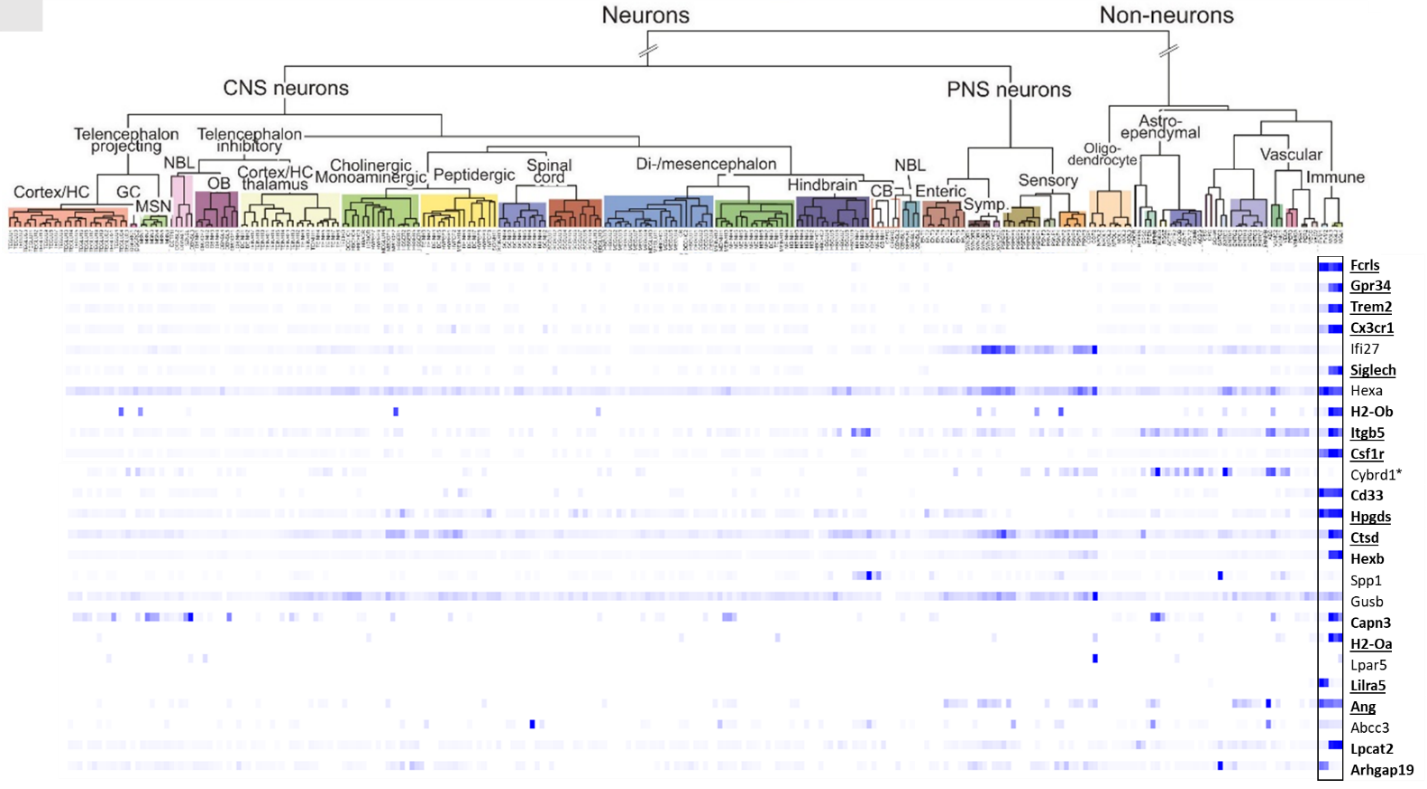


Fig. S4


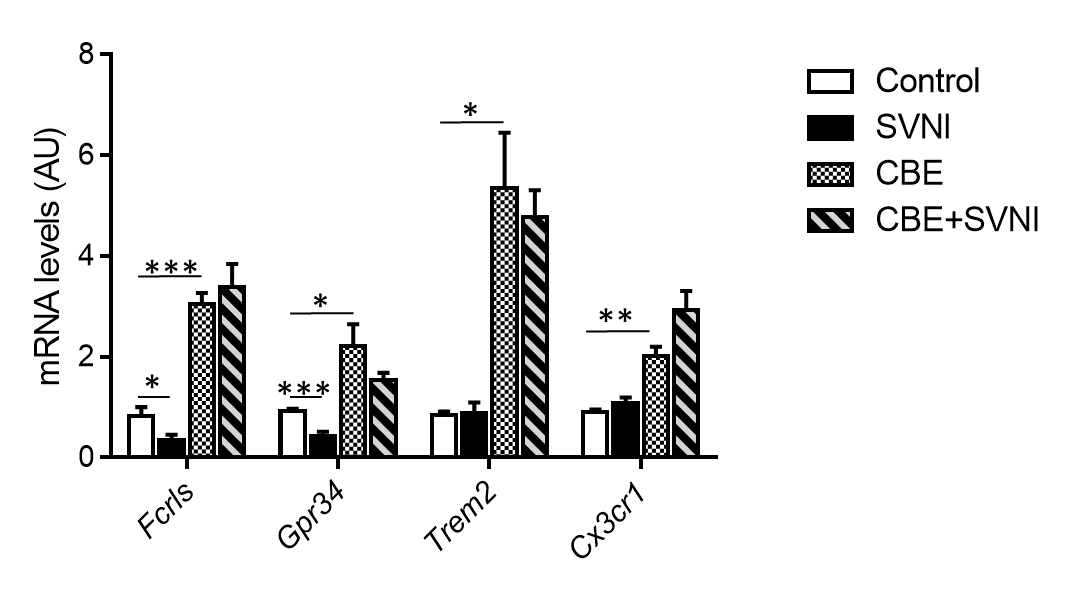


Table S5

| Gene name | Forward primer | Reverse primer |
| --- | --- | --- |
| *Hprt* | TGCTCGAGATGTCATGAAGG | AATCCAGCAGGTCAGCAAAG |
| *Irf7* | CAATGGCTGAAGTGAGGGGG | GACCGAAATGCTTCCAGGGT |
| *Usp18* | CAGGAGTCCCTGATTTGCGT | GGGCTGGACGAAACATCTCA |
| *Fcrls* | CTTGTGAGGCTGAAAACGCC | GCCATTCACCAAACGCACTT |
| *Gpr34* | CCTGGTCTAGGGAGTTTTGGG | GAGCAAAGCCAGCTGTCAAC |
| *Trem2* | TGGCAAAGGAAAGGTGCCAT | ACATGACACCCTCAAGGACTG |
| *Cx3cr1* | CAGGAGAGACCCATCTCCC | GTTGCCTCAACCCCTTTATCT |
